# Study on heart rate recovery index to predict maximum oxygen uptake in healthy adults aged 30 to 60 years old

**DOI:** 10.3389/fphys.2024.1437962

**Published:** 2024-12-24

**Authors:** Guoqing Miao, Qi Yan, Houyuan Zhu, Fantai Li

**Affiliations:** ^1^ China Institute of Sport Science, Beijing, China; ^2^ School of Physical Education, Hebei Normal University, Shijiazhuang, Hebei, China

**Keywords:** heart rate recovery, maximum oxygen uptake, regression equation, healthy people aged 30∼50, cardiorespiratory endurance

## Abstract

**Objective:**

To explore the feasibility of post-exercise heart rate recovery indicators for predicting maximum oxygen uptake (VO2max) in healthy adults aged 30–60 years.

**Methods:**

260 healthy adults who did not perform regular exercise were randomly recruited and divided into a model group (n = 200) and a verification group (n = 60). Measure body fat percentage, weight, height and other indicators, and complete a cardiopulmonary exercise test as required to measure VO2max and heart rate recovery (HRR1, HRR2) in the first and second minutes after exercise. Equations are established through stepwise regression method, and the selected optimal equation is tested for back substitution.

**Results:**

The optimal equation is: $\text{Absolute VO}2\max=-0.528+0.039*\text{weight}-3.463*\text{body fat rate}+0.042*\text{HRR}2-0.180*\text{gender }\left(\text{male}=1,\text{female}=2\right)$ . Analysis of variance, goodness-of-fit test, VIF test, Shapiro-Wilk test, and Durbin-Watson test indicate that the equation is more reliable; Pearson product-moment correlation analysis, paired t test, and Bland-Altman consistency test indicate that the equation is more valid good.

**Conclusion:**

The regression equation established through heart rate recovery after exercise can be used to predict VO2max in healthy adults aged 30–60 years.

## 1 Introduction

Cardiorespiratory endurance is an important indicator that reflects physical health (Writing Committee et al., 2012; Toulouse et al., 2021; Di Prampero, 2003) and is highly correlated with all-cause mortality (Lee et al., 2010), cardiovascular disease mortality (Ren et al., 2020) and the incidence of various tumors (Neto et al., 2019). In 2016, the American Heart Association ranked cardiorespiratory endurance as the fifth vital sign of the human body (Ross et al., 2016). Maximum oxygen uptake (VO2max) is the golden index for evaluating the human cardiorespiratory endurance level (Levine, 2008; Hawkins et al., 2007; Skattebo et al., 2021). VO2max refers to the maximum rate at which an individual can take in and utilize oxygen at maximum exercise intensity. As an important indicator for evaluating cardiorespiratory endurance, it has been widely used in competitive sports, mass sports, etc. (Guazzi et al., 2017; Molinari et al., 2020; Crowley et al., 2022). VO2max testing can be divided into two methods: direct testing and indirect testing. Direct measurement of VO2max is usually done through a cardiopulmonary exercise test. Subjects were required to complete incremental load exercises on a power bike or treadmill (Beltz et al., 2016). This method requires professional equipment and operators, and the test cost is high. It is difficult to popularize it among the public. It requires subjects to reach a state of exhaustion, which involves certain risks. It is also not suitable for the elderly and people with poor physical fitness. The indirect measurement method uses an exercise intensity lower than the maximum load and predicts VO2max from the test results. Although the indirect testing method is not as accurate as the direct testing method, it has attracted much attention due to its relatively low requirements on test sites, equipment, and operators, low economic cost, and easy operation. Indirect testing of VO2max usually uses the subject’s basic physical information and exercise capacity information as independent variables (Hansen et al., 2016; Jalili et al., 2018; Eisenberger et al., 2022).

Heart Rate Recovery (HRR) after exercise refers to the difference between the heart rate at different time periods after exercise and the peak heart rate during exercise. Commonly used measurement times include 1, 2, 3, 4, 5, and 7 min (recorded as HRRt, t corresponds to 1, 2, 3, 4, 5, and 7 min) refers to the individual’s heart rate gradually returning from the level during high-intensity exercise to the heart rate in the resting state after physical activity (Zhu and Lin, 2017). Studies have shown that VO2max tested in cardiopulmonary exercise testing has a positive linear correlation with HRR (Vicente-Campos et al., 2014). The purpose of this study is to establish an evaluation method for maximum oxygen uptake. Using variables such as HRR, body fat percentage, weight, and gender as independent variables, VO2max is evaluated through multiple linear stepwise regression.

## 2 Research objects and research methods

### 2.1 Research objects

Subject inclusion criteria: (1) Age 30–60 years old; (2) No exercise habit; (3) Voluntarily cooperate with the experimental process. Exclusion criteria: (1) suffering from cardiovascular disease and family history of sudden death; (2) suffering from physical pain, trauma, etc. Finally, after screening and data cleaning, the data of 260 subjects (164 males, 96 females) were selected. The above subjects’ data were randomly divided into two groups, of which 200 (120 males, 80 females) were In the model group, 60 people (44 men and 16 women) served as the validation group. The choice to allocate 200 participants to the model group and 60 to the validation group was based on statistical soundness. A larger model group sample size helps ensure the accuracy and stability of the model, while a smaller validation group sample size is sufficient to evaluate the model’s generalizability. This division complies with common rules of thumb and takes into account the effective use of resources and the scientific nature of the model (Table 1).

**TABLE 1:** Basic information on the indicators of the model group and validation group.

Group	Number of people (n)	Age (y)	Gender: Male female
Model group	200	45.6 ± 12.0	120/80
Validation group	60	42.5 ± 10.7	44/16

### 2.2 Research methods

#### 2.2.1 Morphological information measurement

Use the Inbody720 body composition tester to test height, weight, and body fat percentage. The test methods refer to the “National Physical Fitness Measurement Standards (Revised in 2023)” promulgated by the National Physical Fitness Monitoring Center. Calculate the subject’s body mass index (BMI) = weight/height^2^ based on the height and weight tested by Inbody720.

#### 2.2.2 Cardiopulmonary exercise test

Testing equipment: cardiopulmonary exercise function tester (China, Hanya, model SMAX58CE-SP); treadmill (Sweden, RODBY, model RL2000E); heart rate belt (Finland, POLAR, model H10); respiratory mask (United States, HANS RUDOLPH, Model 2797).

The VO2max test plan adopts Bruce’s incremental load treadmill plan. After the tester is ready for the test, he or she puts on the heart rate monitor, respiratory mask, and fastens the safety protection device while standing on the treadmill. The tester once again informed the tester of the testing process and precautions. After the VO2max test, the tester stood still on the treadmill, and HRR1 and HRR2 were measured through the heart rate belt.

Criteria for judging the end of the VO2max test: (1) The heart rate reaches 180 b/min or no longer rises within 2 min; (2) Respiratory quotient ≥1.10; (3) As the exercise intensity increases, the subject’s oxygen uptake plateaus or declines; (4) The subject is unable to maintain the existing exercise intensity despite his best efforts. The equation model group reaches the limit state during the test, and the back-substitution test group stops testing when the test center rate reaches 180 b/min or no longer rises within 2 min.

### 2.3 Statistical analysis

Input the data into spss25.0 statistical software for relevant statistical processing, and the statistical results are expressed in the form of ‾x ± s. Correlation analysis was conducted on the normality of the data and the factors affecting VO2max through the Kolmogorov-Smirnov test and the Pearson correlation coefficient test. Four stepwise regressions were performed, and the best equation for goodness of fit was selected through goodness-of-fit test, VIF test, Durbin-Watson test, etc. The differences between the measured values and the predicted values of the optimal equation were analyzed through paired sample t test, Pearson correlation analysis, and Bland-Altman test. The significance level is P < 0.05, and the very significant level is P < 0.01.

## 3 Results

### 3.1 Body shape test results

The test results are shown in Table 2. The height, weight and BMI of men are significantly higher than that of women (p < 0.01); their body fat rate is significantly lower than that of women (p < 0.01).

**TABLE 2:** Body shape test results.

Index	Male	Female	Overall
Height (cm)	173.92 ± 5.86	164.32 ± 5.77^*^	170.4 ± 7.42
Weight (kg)	72.57 ± 10.02	59.41 ± 9.01^*^	67.75 ± 11.53
BMI (kg/m^2^)	23.97 ± 2.71	21.95 ± 2.79^*^	23.23 ± 2.89
Body fat percentage	0.22 ± 0.05	0.27 ± 0.05^*^	0.24 ± 0.06
Age	45.26 ± 11.98	44.11 ± 11.16	44.80 ± 11.67

Note: *p < 0.01 compared to men.

### 3.2 Cardiopulmonary exercise test results

The test results are shown in Table 3. VO2max is 3.23 ± 0.67 L/min for men and 2.10 ± 0.45 L/min for women; HRR1 is 21.79 ± 5.19 beats/min for men and 19.41 ± 5.61 beats/min for women; HRR2 is 43.30 ± 8.38 beats/min for men. 37.59 ± 9.10 beats/min for women.

**TABLE 3:** Cardiopulmonary exercise test results.

Index	Male	Female	Overall
VO2max (L/min)	3.23 ± 0.67	2.10 ± 0.45	2.81 ± 0.82
HRR1 (beats/min)	21.79 ± 5.19	19.41 ± 5.61	20.84 ± 5.47
HRR2 (beats/min)	43.30 ± 8.38	37.59 ± 9.10	41.02 ± 9.08

### 3.3 Correlation analysis between VO2max and various indicators

Through correlation analysis, it was found that, as shown in Table 4, VO2max has a significant correlation with HRR1, HRR2, body fat rate, height, weight and gender of the overall data (p < 0.01). The correlation between age and VO2max is not significant (p > 0.05).

**TABLE 4:** Correlation between VO2max and various indicators.

	Male	Female	Overall
HRR1 (beats/min)	0.122	0.508^**^	0.273^**^
HRR2 (beats/min)	0.228^**^	0.605^**^	0.398^**^
Body Fat Percentage	0.002	−0.287^**^	−0.318^**^
Height (cm)	−0.659^**^	0.238^*^	−0.449^**^
Weight (kg)	0.769^**^	0.153	0.769^**^
BMI (kg/m^2^)	0.728^**^	0.036	0.675^**^
Age	−0.046	−0.133	−0.01
Gender			−0.596^**^

Note: * represents p < 0.05; ** represents p < 0.01.

### 3.4 Establishment of regression equation

Through stepwise regression analysis, HRR1, HRR2, body fat percentage, height, weight, BMI and gender were brought into the regression equation. The regression analysis results are shown in Tables 5, 6.

**TABLE 5:** Summary of regression equations.

Equation	R	R2	Adjusted R-square	Standard error	Durbin-Watson
1	0.769a	0.592	0.590	0.708	1.511
2	0.870b	0.758	0.756	0.546	1.581
3	0.922c	0.850	0.848	0.431	1.625
4	0.924d	0.853	0.851	0.427	1.624

Note: a. Predictor variables: (constant), body weight; b. Predictor variables: (constant), body weight, body fat rate; c. Predictor variables: (constant) body weight, body fat rate, HRR2; d. Predictor variables: (constant) weight, body fat percentage, HRR2, gender.

**TABLE 6:** Correlation coefficient in Equation 4.

Index	B	Standard error	β	t	P	Tolerance	VIF
Constant	−0.528	0.267		−1.983	0.048		
Weight	0.039	0.001	0.790	27.673	0.000	0.706	1.416
Body Fat Percentage	−3.463	0.593	−0.188	−5.840	0.000	0.555	1.800
HRR2	0.042	0.003	0.348	12.308	0.000	0.718	1.392
Gender	−0.180	0.074	−0.079	−2.429	0.016	0.550	1.817

According to the regression results, the estimated VO2max equation can be finally obtained:

AbsoluteVO2⁡max=0.222+0.038*body weight (1)

Absolute VO2⁡max=1.853+0.041*body weight−7.553*body fat rate (2)

Absolute VO2⁡max=−0.778+0.041*weight−4.158*body fat rate+0.043*HRR2 (3)

$$\text{Absolute VO}2\max=-0.528+0.039*\text{weight}-3.463*\text{body fat rate}+0.042*\text{HRR}2-0.180*\text{gender }\left(\text{male}=1,\text{female}=2\right)$$ (4)

As (Equation 1) can be seen from Table 5, the correlation coefficient (R) of model 1 is 0.769, indicating that there is a moderate positive correlation between VO2max and body weight. The coefficient of determination (R2) is 0.592, indicating that this model can explain 59.2% of VO2max. The adjusted coefficient of determination (adjusted R2) is 0.590. Taking into account the influence of the number of independent variables and sample size in the model, the explanatory power of the model has improved. The standard error is 0.708 and the Durbin-Watson test is 1.511.

The correlation coefficient (R) of model 2 is 0.870, indicating that there is a strong positive correlation between VO2max (Equation 2), body weight and body fat percentage. The coefficient of determination (R2) is 0.758, indicating that this model can explain 75.8% of VO2max. The adjusted coefficient of determination (adjusted R2) is 0.756. Taking into account the influence of the number of independent variables and sample size in the model, the explanatory power of the model has improved. The standard error is 0.546 and the Durbin-Watson test is 1.581.

The correlation coefficient (R) of model 3 is 0.922, indicating (Equation 3) that there is a strong positive correlation between VO2max and body weight, body fat rate and HRR2. The coefficient of determination (R2) is 0.850, indicating that this model can explain 85.0% of VO2max. The adjusted coefficient of determination (adjusted R2) is 0.848. Taking into account the influence of the number of independent variables and sample size in the model, the explanatory power of the model has improved. The standard error is 0.431 and the Durbin-Watson test is 1.625.

The correlation coefficient (R) of model 4 is 0.924 (Equation 4), indicating that there is a strong positive correlation between VO2max and body weight, body fat rate and HRR2. The coefficient of determination (R2) is 0.853, indicating that this model can explain 85.3% of VO2max. The adjusted coefficient of determination (adjusted R2) is 0.851. Taking into account the influence of the number of independent variables and sample size in the model, the explanatory power of the model has improved. The standard error is 0.427 and the Durbin-Watson test is 1.624.

In summary, Model 4 has strong explanatory power, with higher R2 and higher adjusted R2, taking into account the effects of weight, body fat rate, HRR2 and gender. The standard error is small. The Durbin-Watson test is close to the ideal range, indicating that the residuals in this model are independent of each other. Comprehensive analysis results show that body weight, body fat percentage, HRR2 and gender have a significant impact on the ability to explain VO2max. The explanatory power of these models is relatively strong, the standard errors are small, and the research results have certain reference value.

### 3.5 Backward elimination test

Substitute various indicators of the verification group (n = 60) into the optimal equation to predict the VO2max value and analyze and compare it with the measured VO2max value. Through paired sample t test and Pearson correlation analysis test, the test results show that the measured value and predicted value The difference is not significant (p > 0.05) and there is a high positive correlation (r = 0.889, p < 0.01) (Table 7).

**TABLE 7:** Comparative analysis of actual measured values and predicted values.

	Measured values	Predicted values	Paired samples T-Test		Pearson correlation test	
	(L/min)	(L/min)	t	p	r	p
Data	2.893 ± 0.752	2.931 ± 0.688	−0.832	0.408	0.886	0.000

## 4 Discussion

Using stepwise regression analysis, four regression equations for inferring VO2max were established. Test through goodness of fit test, VIF test, Durbin-Watson and other methods. The best equation is adopted based on the test results. The best equation is $\text{Absolute VO}2\max=-0.528+0.039*\text{weight}-3.463*\text{body fat rate}+0.042*\text{HRR}2-0.180*\text{gender }\left(\text{male}=1,\text{female}=2\right)$ . In order to further explain the degree of explanation of the dependent variable by the independent variables in the equation, a goodness-of-fit test was performed on the regression equation. The test results showed: R = 0.924, R2 = 0.853, adjusted R2 = 0.851, standard error = 0.427, indicating that the fitting degree of the equation is good. The multicollinearity of the equation is one of the important factors that affects the accuracy of the prediction results of the equation. When VIF > 5 and tolerance <0.1, it indicates that the equation has multicollinearity problems. In this study, the VIF and tolerance of the optimal equation selected through stepwise regression analysis were 1.817 and 0.550 respectively, indicating that there is no multicollinearity problem among the independent variables in the equation. In addition to meeting the above conditions, the residuals of the equation must also meet normality and independence. The normality and independence of the residuals of the regression equation were tested using the Shapiro-Wilk and Durbin-Watson tests respectively. The Shapiro-Wilk test result shows that the residuals of the equation conform to the normal distribution, and the Durbin-Watson test result is 1.624, indicating that the residuals of the equation are independent. The normality of residuals affects the validity of parameter estimation and hypothesis testing of regression models. The independence of residuals affects the stability of the model, its predictive power, and the reliability of hypothesis testing. Therefore, ensuring that the residuals meet the assumptions of normality and independence is an important step in the overall reliability of the regression model.

The independent variables included in the optimal equation of this study are weight, body fat percentage, HRR2 and gender. There is a significant positive correlation between absolute VO2max and body weight. Onetti-Onetti et al. (2020) proposed that body weight is an important influencing factor for VO2max. This is consistent with the research results of Tangkudung et al. (2020). In this study, body weight was used as the natural factor. Variables are included in the regression equation. There is a significant negative correlation between body fat rate and absolute VO2max, This means that a lower body fat percentage is beneficial to an individual’s VO2max. Kai et al. (2024) used 48 subjects as the research subjects, and the results showed that body fat rate is beneficial to the individual’s aerobic exercise ability. There was a significant negative correlation between fat percentage and absolute VO2max (r = −0.55, p < 0.001), which is consistent with the research results of Mondal and Mishra (2017). Absolute VO2max has a significant positive correlation with HRR2. As heart rate recovery ability increases, aerobic exercise capacity will also be enhanced. Yifan et al. (2014) divided HRR242bmp into one group, and divided HRR2 < 42bmp into another group. One group, a comparative analysis of the VO2max and other indicators of the two groups found that the VO2max of the high HRR2 group was significantly higher than the other group. Gender is an important influencing factor on VO2max. Research by Xiaoyun and Meng (2005) and others pointed out that differences in gender will cause differences in VO2max. Women are usually lower than men, which is related to the fact that men have higher heart volume, hemoglobin content and cardiac output than women (Santisteban et al., 2022). The study by Wiebe et al. (1998) also found that women’s VO2max is significantly lower than men.

**FIGURE 1:**
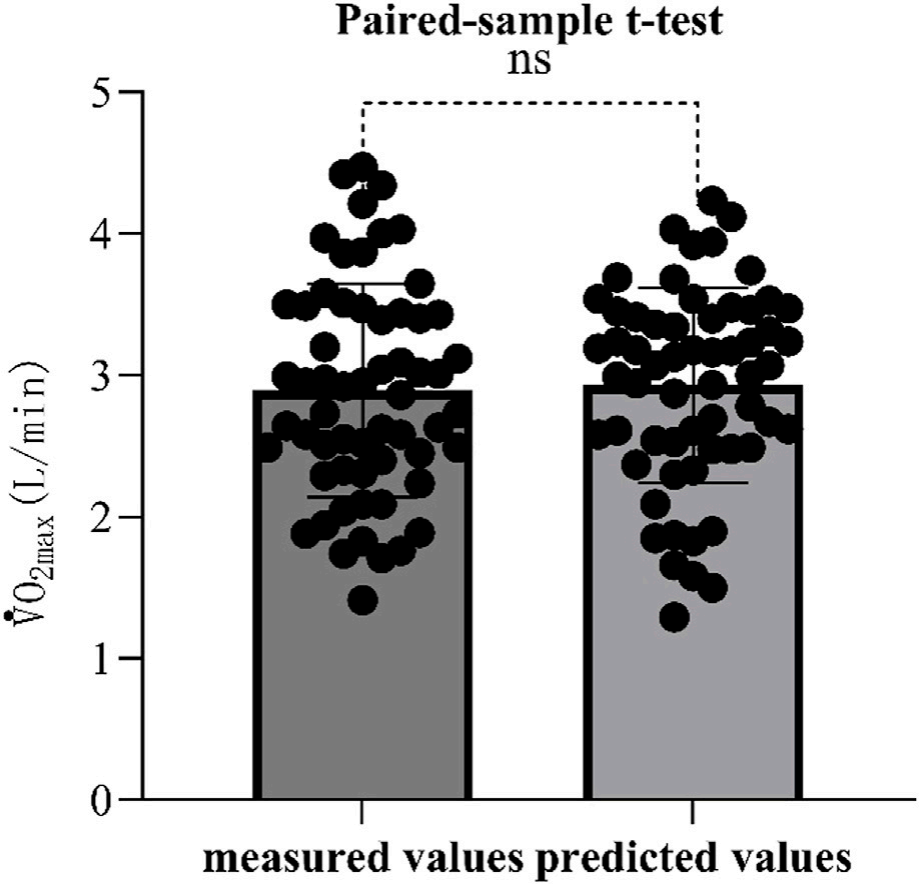
Paired sample t-test of measured values and predicted values. Note: ns means p > 0.05, no significant difference.

**FIGURE 2:**
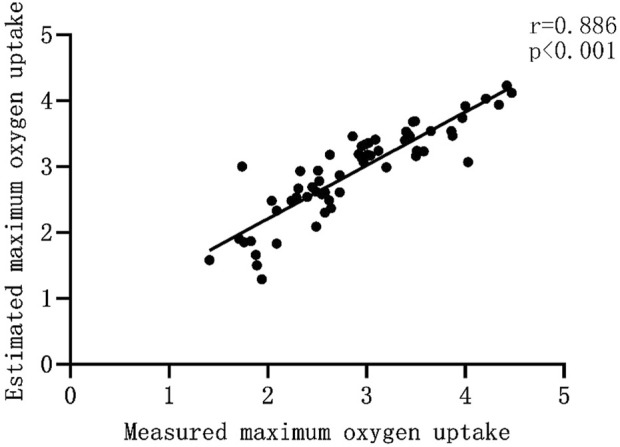
Pearson correlation analysis between actual measured values and predicted values.

**FIGURE 3:**
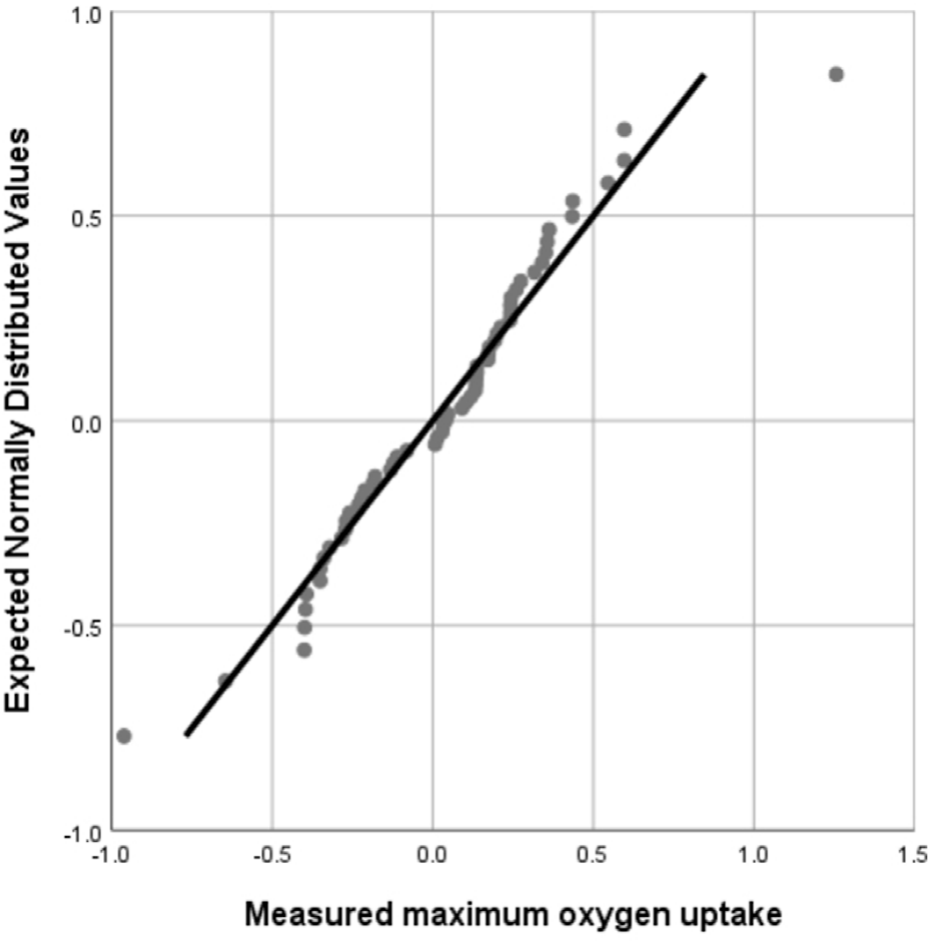
Normal Q-Q plot of the difference between actual measured value and predicted value.

**FIGURE 4:**
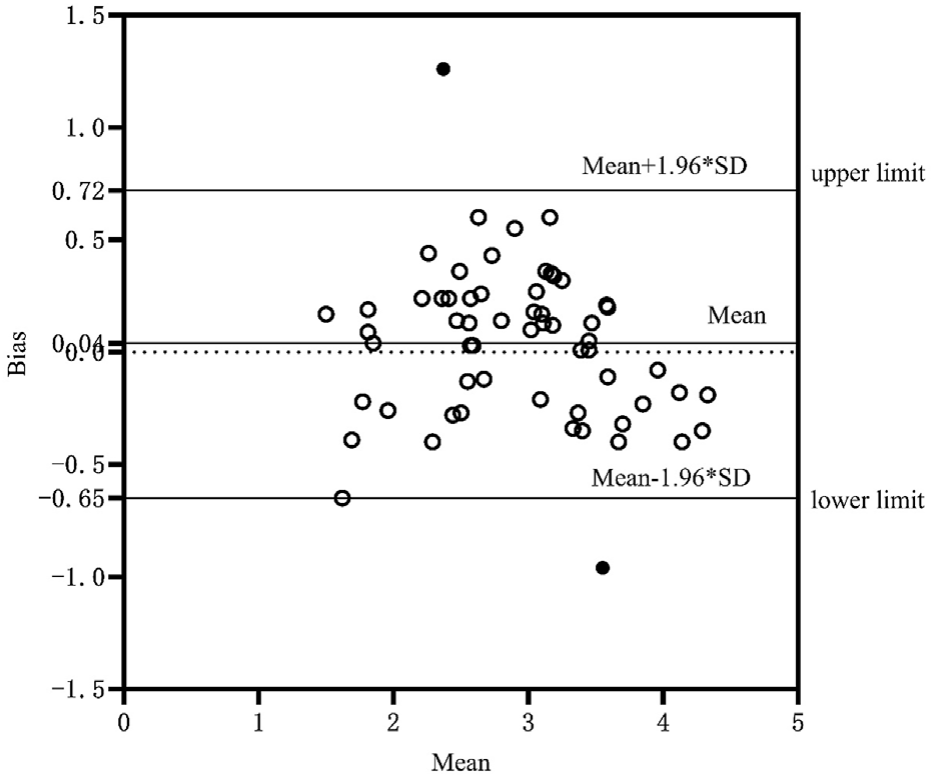
Mean systematic error between measured values and predicted values.

The validity of the equation is tested by substituting the data of the validation group into the model group, and the measured and predicted values of the validation group are analyzed using paired sample t-test, Pearson correlation analysis and Bland Altman test. Bland-Altman analysis is a method used to evaluate the consistency between two measurement methods. It is usually used to compare the deviation and consistency of two measurement methods (Gerke, 2020) and is intuitively reflected through graphics. In this study, the value calculated by the optimal equation was slightly lower than the measured value, which may be related to the individual differences of the subjects. At the same time, the paired sample t test showed that there was no significant difference between the measured value and the predicted value (Figure 1), Pearson product-moment correlation analysis suggests that the two are highly correlated (Figure 2). The normality test was performed on the difference between the measured value and the predicted value in the verification group, and the results showed that the difference was normally distributed (Figure 3). The actual measured values and predicted values of the validation group were further evaluated. Through the Bland-Altman consistency test, it was found that the mean VO2max difference of 58 of the 60 subjects in the validation group was within the Mean ± 1.96*SD interval. Only two subjects had mean differences outside the Mean ± 1.96*SD interval (Figure 4), which shows that the optimal equation used in this study has good validity.

Although the equation has included some major factors such as weight, body fat percentage, HRR2 and gender, there are also some potential confounding variables that may not have been taken into account, such as age, height, training status, genetic factors and lifestyle factors. These potential confounding variables may affect the accuracy of VO2max prediction. In practical applications, it is recommended to further examine and control these potential confounding variables to improve the reliability and validity of the model.

HRR is an important indicator of cardiovascular health (Dimkpa, 2009), which reflects the heart’s ability to recover from a high-intensity state to a resting state after exercise and reflects the body’s ability to adapt to exercise load. HRR not only has the feasibility of evaluating aerobic capacity, but has also been proven to be an effective independent indicator for predicting the occurrence of cardiovascular disease and cardiovascular events (Cole et al., 2000; Cole and Lauer, 1999; Nishime et al., 2000). This is one of the reasons why this study uses HRR as a predictor of VO2max. In addition, and most importantly, HRR measurement is relatively simple, usually just recording the heart rate drop after high-intensity exercise, without the need for complex equipment. This makes the inferred VO2max equation established through HRR highly operable, practical and easy to popularize.

## 5 Conclusion

The prediction equation established in this study is: Absolute VO2⁡max=−0.528+0.039*weight−3.463*body fat rate+0.042*HRR2−0.180*gender (where HRR2 is the peak heart rate during exercise-the heart rate within 2 minutes after the end of exercise); Gender: male = 1, female = 2, HRR2 measurement is relatively simple and convenient, and the reliability and validity test of this equation is good, and it is suitable for promotion and use in large sample populations.

## Data Availability

The raw data supporting the conclusions of this article will be made available by the authors, without undue reservation.
